# How Do the Valency and Radii of Cations Affect the Rheological Properties of Aqueous Solutions of Zwitterionic and Anionic Surfactant Mixtures?

**DOI:** 10.1021/acs.langmuir.4c04689

**Published:** 2025-01-29

**Authors:** Ewelina Warmbier-Wytykowska, Ashley Peter Williams, Jacek Rozanski, Peter Fischer, Viviane Lutz-Bueno, Stephan Handschin, Laura Baraldi, Jarosław Warmbier, Patrycja Wagner, Sylwia Różańska

**Affiliations:** † Institute of Chemical Technology and Engineering, Faculty of Chemical Technology, Poznan University of Technology, ul. Berdychowo 4, PL 60-965 Poznan, Poland; ‡ Laboratory for Neutron Scattering and Imaging, Paul Scherrer Institut, 5232 Villigen, Switzerland; § Institute of Food, Nutrition and Health, ETH Zürich, Schmelzbergstrasse 7, 8092 Zürich, Switzerland; ∥ Scientific Center for Optical and Electron Microscopy, ETH Zürich, Auguste-Piccard-Hof 1, 8093 Zürich, Switzerland; ⊥ Faculty of Control, Robotics and Electrical Engineering, Division of Control and Robotics, Poznan University of Technology, ul. Piotrowo 3a, 60-965 Poznan, Poland

## Abstract

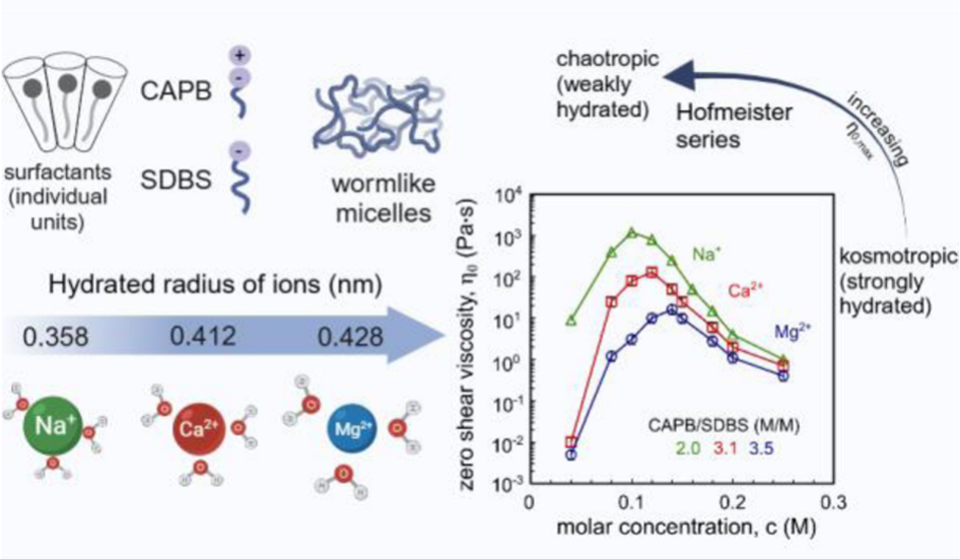

Despite extensive research on the use of salts to enhance micellar growth, numerous questions remain regarding the impact of ionic exchange and molecular structure on charge neutralization. This study looks into how certain cations (Na^+^, Ca^2+^, and Mg^2+^) affect the structure of a cocamidopropyl betaine CAPB and sodium dodecylbenzenesulfonate SDBS surfactant mixture, aiming toward applications in targeted delivery systems. The mixture consists of a zwitterionic surfactant, cocamidopropyl betaine (CAPB), and an anionic surfactant, sodium dodecylbenzenesulfonate (SDBS), combined in varying molar ratios at a total concentration of 200 mM. We characterized the macroscale properties through rheological measurements and obtained detailed structural insights using small-angle X-ray scattering (SAXS) and cryogenic electron microscopy (cryo-EM). The findings reveal that increasing the concentration of cations in the CAPB/SDBS mixture induces the formation of peaks in the zero-shear viscosity as a function of salt concentration (salt curve). Analysis through cryo-EM and SAXS showed that these viscosity peaks are related to the change of micellar assemblies from entangled worm-like micelles to branched worm-like micelles and then to bilayer structures (vesicles). The specific cation concentration at which the zero-shear viscosity peak occurs, as well as the maximum viscosity, is strongly influenced by the type of cation present in the CAPB/SDBS solutions, a phenomenon explained by the Hofmeister series. Notably, the differing affinities of cations for the carboxylate COO^–^ and sulfite SO^3–^ groups and the partial dehydration of micelles contribute to the lower concentration of magnesium cations required to reach the viscosity peak compared to calcium cations.

## 1 Introduction

Surfactants, also called surface-active agents (SAAs), are very important to everyday life, as shown by the fact that they are produced in large amounts around the world and are used in many different ways but also can pose a threat to the environment.^1−3^ Consequently, there is a discernible trend toward the exploration of environmentally friendly surfactant solutions, including those derived from natural sources. SAAs possess a multitude of remarkable properties, including the ability to reduce surface tension and form micelles. Besides the well-known spherical micelles, worm-like micelles (WLMs), which are long micelles, also called living polymers,^4−6^ sponge phases, lamellae phases, or vesicles can be formed. The term “living polymers” indicates that fluids containing worm-like micelles exhibit rheological properties similar to polymer solutions. However, there is one fundamental difference: while polymers are connected by covalent bonds between monomers, the molecules in micelles are held together by physical interactions. Therefore, they are termed “living” polymers, meaning systems that undergo continuous disintegration and reformation. The rheological properties of surfactant solutions are intricately linked to the nature of the resulting micellar structures. Viscoelastic fluids characterized by elevated viscosity levels can be achieved through the intertwining of WLMs formed within surfactant solutions, culminating in the development of a spatial network.^4,5^ Regularly, the entangled network undergoes a transformation into a branched network configuration.

Elucidating the mechanisms of micellar association is a complex task, especially when considering the impact of additives such as metal cations and chelates. Simple salts are a classic example of initiators that transform spherical micelles into worm-like micelles, but their effectiveness is limited by concentration constraints. For instance, the addition of simple salts to solutions of anionic surfactants (most commonly NaCl and KCl),^5−7^ hydrotropes,^4,8^ or cosurfactants^8−10^ can induce the formation of WLM. In cosmetics and home chemistry, WLM is made by mixing the right kinds of surfactants together, such as anionic and cationic surfactants,^9,11−14^ anionic and zwitterionic surfactants,^7−19^ or anionic and nonionic surfactants.^20−24^ Even though the role of salts to facilitate micellar growth is studied for many years, the way how ionic exchange and molecular structure affect charge neutralization is not fully understood. The situation becomes particularly challenging when considering ions of different valencies, which entails variations in ionic radii. The morphological transitions of surfactant aggregates and their resulting viscoelasticity directly relate to this.

The relationship between surfactant molecules is traditionally described using the packing parameter.^25,26^ According to the proposal by Israelachvili et al.,^25^ the shape of the micelles formed in the solution depends on the value of the packing parameter
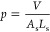
1where *V* is the volume of the hydrophobic tail, *A*_S_ the effective surface area occupied by the polar head on the micelle surface, and *L*_S_ the length of the hydrophobic tail, defined as the maximum length of the stretched chain. If *p* is less than 1/3, the solution will form spherical micelles, for 1/3 < *p* < 1/2 worm-like micelles, 1/2 < *p* < 1 vesicle micelles, *p* ≈ 1 flat lamellar structure, and a reverse micelles if *p* is greater than 1. The addition of simple salts to solutions of anionic surfactants results in the screening of the charged heads of surfactant molecules, thereby reducing the repulsion between them. This leads to a decrease in the effective surface area *A*_s_ and an increase in the packing parameter value, consequently causing changes in the shape of the micelles.^26−29^

There are commercially available surfactants, which can act as possible candidates for other uses such as delivery systems or gel-like fertilizers in agrochemicals. Betaines like cocamidopropyl betaine (CAPB), a class of zwitterionic (amphoteric) biobased surfactants, are synthesized from coconut pulp and may potentially serve as biodegradable yet environmentally friendly counterparts to synthetic surfactants.^30^ CAPB is commonly utilized as an ingredient in cosmetics,^7,30^ household applications,^7,30,31^ and in flow resistance reduction.^12,32^ It is typically marketed as a 30 wt % solution containing approximately 2 wt % sodium chloride. When combined with anionic surfactants, it forms viscoelastic fluids whose rheological properties strongly depend on pH and temperature.^15,16^ We suggest combining it with sodium dodecylbenzenesulfonate (SDBS), a anionic surfactant, which is commonly used in personal care products and recognized as a safe substance.^33^ One example of its application in the sustainable world is the use of SDBS in fabricating amperometric biosensors for glucose.^34^ Recent research indicates that the rheological properties of the CAPB/SDBS mixture also significantly rely on the concentration of sodium chloride.^7^ The viscosity of CAPB/SDBS mixture solutions is slightly higher than that of pure water and the formation of worm-like micellar network required the introduction of sodium cations into the solution. The influence of sodium chloride on viscosity is additionally dependent on the molar ratio of CAPB/SDBS.^7^

As previously mentioned, simple salts like sodium chloride play a critical role in promoting anisotropic growth and forming viscoelastic surfactant systems composed of worm-like micelles.^4−7,34−37^ The addition of counterions significantly impacts the rheological properties of surfactant systems. Although the effects of monovalent ions have been extensively studied, further investigation into multivalent ions in both organic and inorganic salts remains valuable. These ions help screen the electrostatic repulsion between charged surfactant headgroups. Key factors such as ion valency, charge, size, and hydrophobicity play critical roles in determining their influence on the micellar microstructure. By correlating these different factors, we can attempt to predict the impact of specific ions. For cationic systems, it has been observed that with increasing electronegativity of the ions, micelle surface neutralization increases, which in turn leads to greater micelle length.^38,39^ Mütze et al.^38^ demonstrated that salt counterion electronegativity correlates with zero-shear viscosity increases, particularly for salicylate ions. Other findings on the effects of salts have also been reported for different ionic systems.^8,40,41^

The Hofmeister series (HS) provides a framework for arranging ions according to their water affinity, enabling predictions regarding the preferential interactions of oppositely charged ions based on characteristics like ionic radius, charge, and charge density.^42−47^ Generally, ions with smaller radii and higher charges exhibit greater charge density, categorizing them as water-structuring kosmotropes. Such ions have larger hydration shells, increasing the hydrated ion radius. This increased radius, in turn, lowers charge density, resulting in weaker interactions with oppositely charged ions in aqueous solutions, including those in surfactant systems. In this work, we focus on cation effects, which, according to the HS, are ordered as follows: N(CH_3_)^4+^ > NH_4_^+^ > Cs^+^ > Rb^+^ > Na^+^ > Li^+^ > Ca^2+^ > Mg^2+^ > Zn^2+^ > Ba^2+^.

Research on the influence of multivalent cations on the rheological properties of zwitterionic and anionic surfactant systems remains limited.^19,48^ Qiao et al.^19^ indicates that the valence of the introduced cations influences the zero-shear viscosity of aqueous solutions containing a mixture of sodium dodecyl sulfate (SDS) and tetradecyldimethylammoniumpropanesulfonate (TPS). Notably, the concentrations of multivalent ions (Al^3+^, Ca^2+^, Mg^2+^, and Zn^2+^) required to elicit a marked increase in the viscosity of SDS/TPS solutions were significantly lower than those of the monovalent sodium cation Na^+^. Mitrinova et al.^48^ conducted research on the influence of mono- and multivalent cations (Na^+^, NH^4+^, K^+^, Mg^2+^, Ca^2+^, and Al^3+^) on the rheological properties of aqueous solutions containing a mixture of CAPB and SLES (sodium lauryl ether sulfate) equal to 300 mM and a fixed 2:1 weight ratio.

The objective of the current research is to elucidate the relationship between the molecular structure of selected surfactants and the micellar microstructure responsible for the rheological properties of advanced viscoelastic surfactant mixtures with the addition of selected metal ions. The study seeks to determine the correlation between the concentration of specific metal cations in the solution and the type and shape of the resulting micelles. This study provides a closer look at the mechanisms of self-assembly with cation additives using techniques such as cryogenic electron microscopy (cryo-EM) and small-angle X-ray scattering (SAXS). No previous study has combined these techniques to investigate the influence of metal ions on anionic and zwitterionic systems. This research will lead to the development of innovative delivery systems based on viscoelastic surfactant mixtures, paving the way for further analyses.

## 2 Materials and Methods

### 2.1 Materials

**Figure 1:**
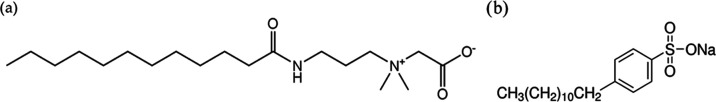
Structure of (a) cocamidopropyl betaine (CAPB) and (b) sodium dodecylbenzenesulfonate (SDBS) (drawings created by ChemSketch).

Cocamidopropyl betaine (CAPB, Figure 1a), with a molecular weight of 342.288 g/mol, was sourced from PCC Exol (Poland) and is marketed under the trade name Rokamina K30. It contained 30 wt % active substance and no more than 6 wt % sodium chloride (NaCl). Sodium dodecylbenzenesulfonate (SDBS, Figure 1b) was acquired from Sigma-Aldrich with an average molecular weight of 348.48 g/mol. Sodium chloride (NaCl, *M* = 58.44 g/mol), calcium chloride (CaCl_2_, *M* = 110.98 g/mol), and magnesium chloride (MgCl_2_, *M* = 95.211 g/mol) were added to determine the Na^+^, Ca^2+^, and Mg^2+^ ion concentrations in the surfactant solutions. All salts were obtained from Chempur (Poland).

### 2.2 Preparation of Solutions

A purification process was employed to remove NaCl from CAPB. All aqueous surfactant solutions were prepared in the same manner. The initial CAPB product was heated at 60 °C to evaporate the water, and the remaining dry residue was then dissolved in chloroform. To remove NaCl crystals, the solution was filtered using paper. Chloroform was subsequently removed from the filtrate through evaporation, and the remaining dry mass was dissolved again in water. The required amounts of SDBS and CAPB were initially dissolved in a small quantity of distilled water. To the CAPB solution, a specific amount of salt (up to 0.3 M) was added. Subsequently, the prepared pretest mixtures were combined, with SDBS solution always being added to the CAPB/salt mixture. The surfactant mixtures were then diluted with distilled water to reach the target concentrations. Systems with varying CAPB/SDBS molar ratios (ranging from 1.5 to 8.0) were prepared for testing, while maintaining a constant total surfactant concentration of 0.2 M. The surfactant solutions were stirred at 50 °C for 4 h using a magnetic stirrer and then left to stand for 24 h. No air bubbles were observed in the solutions.

### 2.3 Rheological Measurements (Shear and Extensional Flow)

Rheological measurements in shear flow were performed with a Physica MCR 501 rotational rheometer (Anton Paar, Austria), concentric cylinder geometrics CC27/T200/AL with a gap diameter of 1.0844 mm. Small amplitude oscillatory shear measurements were performed in the linear viscoelastic regime determined previously by oscillatory strain sweep measurements, in the frequency region from 0.01 to 100 rad/s at strain amplitude γ_0_ = 0.1%. The parameters obtained from the oscillatory test data were: storage modulus *G*′ and loss modulus *G*″. A temperature-control unit (Peltier plate) equipped the measuring device, ensuring temperature control over an extended period. All measurements were conducted at a temperature of 20 °C.

Rheological measurements in extensional flow were conducted using a HAAKE CaBER 1 rheometer (Capillary Breakup Extensional Rheometer, Thermo Fisher Scientific, Germany). The sample was positioned between two parallel plates, each with a diameter of 4 mm, and subjected to an initial step strain of 50 ms duration. This deformation created a liquid bridge between the cylindrical fixtures, initiating a self-driven uniaxial extensional flow that culminated in filament breakup. The resulting apparent transient extensional viscosity function provided insight into the fluid’s response to the axial step strain. Measurements were performed with an initial gap of 2 mm, a final filament height of 5.08 mm, and under ambient temperature conditions.

### 2.4 Small-Angle X-ray Scattering (SAXS)

Small-angle X-ray scattering (SAXS) data were acquired using a Bruker AXS Micro system featuring a microfocused X-ray source operating at 50 kV and 1000 μA. The Cu Kα radiation with a wavelength of 1.5418 Å was directed through a 2D Kratky Collimator and a 2D Pilatus 100 K detector was used to capture the data. The scattering vector *q* = (4 π/λ) sinΘ, where 2Θ represents the scattering angle, was calibrated with silver behenate. The samples were loaded into a 2 mm quartz capillary and sealed with Bondic glue to prevent damage or contamination. After the capillary was filled and sealed, it was carefully placed into the sample holder of the Bruker SAXS instrument for measurement. Data processing and azimuthal averaging were performed using the Saxsgui software to obtain one-dimensional intensity versus scattering vector *q* in the range of 0.004 to 0.4 Å^–1^. SAXS measurements were conducted at 20 °C, with a 30 min data collection period.

### 2.5 Cryogenic Electron Microscopy (Cryo-EM)

The micellar microstructure was examined by cryogenic transmission electron microscopy (cryo-EM). Sample preparation was conducted in a controlled-environment vitrification system (Vitrobot Mark IV, Thermo Fisher Scientific) maintained at 22 °C and 100% humidity. Then the sample (3 μL) was deposited onto hydrophilized lacey carbon-coated copper grids (EMS) and excess liquid was removed using filter paper to create a thin film across the grid mesh. The grids were subsequently vitrified by plunging into a liquid ethane-propane mixture cooled with liquid nitrogen. For automated loading in the cryo-EM, the vitrified grids were clipped into AutoGrid sample carriers (Thermo Fisher Scientific). Data acquisition was performed on a TFS Titan Krios (Thermo Fisher Scientific) operating at an acceleration voltage of 300 kV. The microscope was equipped with a Gatan Quantum-LS Energy Filter (GIF) and a Gatan K2 Summit direct electron detector. Imaging of the vitrified specimens was carried out in energy-filtered TEM (EFTEM) mode utilizing the TFS EPU software and the K2 camera in linear mode.

### 2.6 Surface Tension

Surface tension measurements were performed using a K12 tensiometer (Krüss GmbH, Hamburg, Germany). The du Nouy ring method was used, in which a platinum–iridium ring is immersed in the liquid and then pulled up at a constant speed (0.1 m s^–1^) until it detaches from the sample. Each measurement was repeated three times.

### 2.7 Statistical Analysis

The values of the shear and extensional viscosities presented in the graphs and tables are the arithmetic means of five repetitions. Confidence intervals were calculated using a 95% confidence level.

## 3 Results and Discussion

### 3.1 Zero-Shear Viscosity

**Figure 2:**
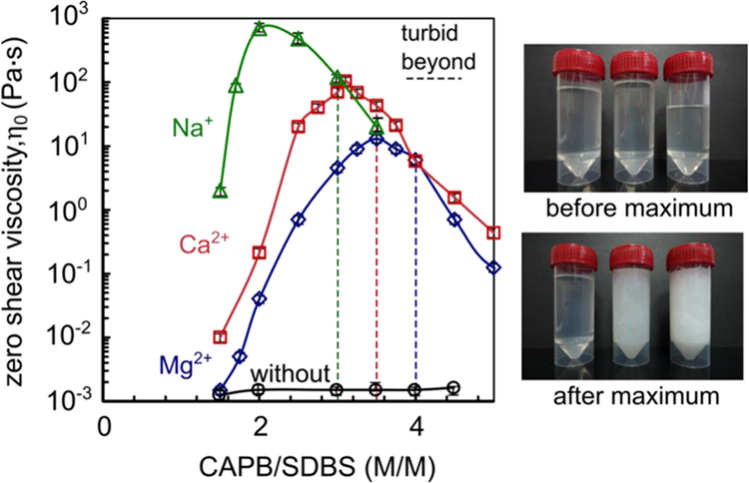
Dependence of zero-shear viscosity on the molar ratio of CAPB/SDBS in aqueous solutions with the addition of NaCl, MgCl_2_, and CaCl_2_ (0.1 M). The images on the right represent samples with Mg^2+^, but the same tendency was observed for other types. A line has been marked indicating the threshold at which the samples become turbid, with representative images displayed on the right.

The influence of monovalent Na^+^ and divalent Ca^2+^ and Mg^2+^ ions on the viscous and elastic properties of CAPB/SDBS solutions was investigated through shear and oscillatory rheology. This study addressed two key aspects: The effect of (i) the molar ratio of the surfactants on the ion concentration and the (ii) impact of varying ion concentrations while maintaining a constant molar ratio of the surfactants. The first aspect focused on different molar ratios. Here, ion concentrations (100 mM) and the total concentration of surfactants (200 mM) were kept constant and the solutions varied in the molar ratio of CAPB to SDBS. Based on the flow curves (Figures S1 and S2 in Supporting Information), zero-shear viscosity values were determined and are presented as a function of the CAPB/SDBS molar ratio in Figure 2. The addition of ions significantly increases the zero-shear viscosity with an increasing molar ratio of CAPB. A distinct maximum in zero-shear viscosity was observed, indicating structural transformations within the micellar network. The viscosity peaks for solutions containing Na^+^, Ca^2+^, and Mg^2+^ ions occur at CAPB/SDBS molar ratios of 2.0, 3.1, and 3.5, respectively. Furthermore, there are notable differences in the zero-shear viscosity values at these peaks, depending on the type of ion present. Specifically, the maximum zero-shear viscosity for the solution with sodium cations is approximately ten times higher than that for solutions with calcium cations and about one hundred times higher than that for solutions with magnesium cations.

The occurrence of a viscosity peak with varying surfactant molar ratios has been documented in the literature for various systems, including zwitterionic/anionic surfactants,^5,7,15−20^ cationic/anionic surfactants,^10,11,13,14^ and nonionic/anionic surfactants.^21−23,49^ For the CAPB/SDBS mixture, such observations were reported by Różańska et al.,^7^ who noted varying salt concentrations in the surfactant solutions and demonstrated that the presence of sodium cations is essential for the formation of entangled worm-like micelles (WLM) in CAPB/SDBS mixtures as well as that the effect of salts on WLM formation is closely linked to the molar ratio of CAPB to SDBS in the solution. According to the data presented in Figure 2, the CAPB/SDBS molar ratio at which the viscosity peak occurs is also dependent on the type of cation present in the solution. Figure 2 includes additional images of CAPB/SDBS samples with Mg^2+^, captured both below and above the viscosity peak. The surfactant mixture solutions remained clear up to a CAPB/SDBS molar ratio of 4, whereas samples with a molar ratio of 5 or higher exhibited turbidity. This turbidity suggests the formation of layered micelles such as vesicular or lamellar structures.

**Figure 3:**
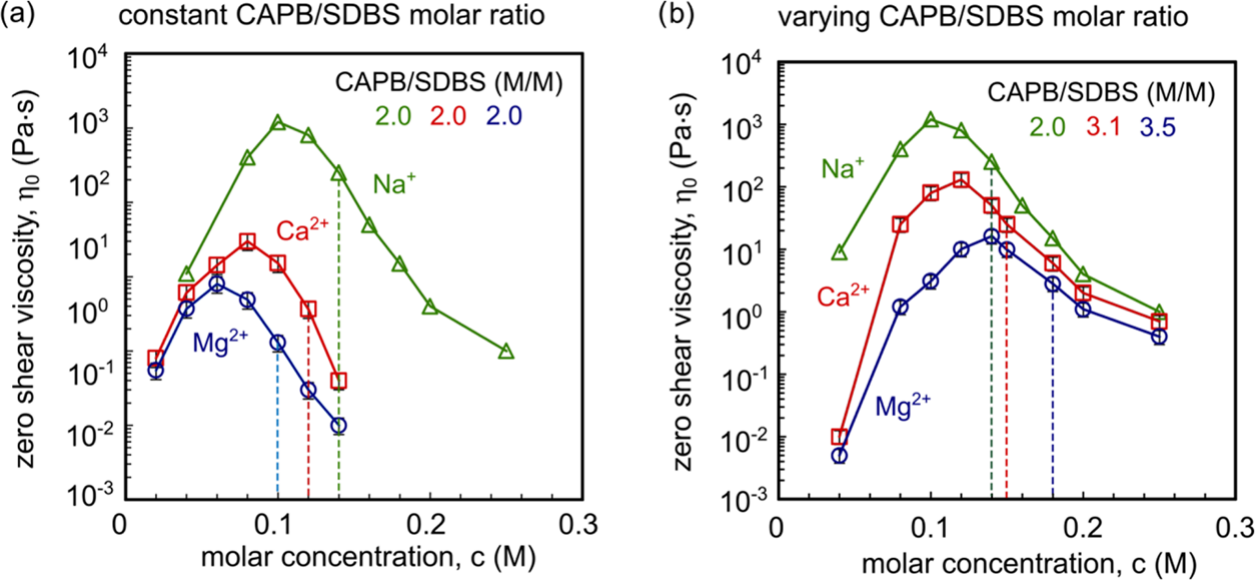
Influence of metal ions on the zero-shear viscosity of different aqueous CAPB/SDBS solutions. (a) Fixed molar ratio of 2.0 for Na^+^, Ca^2+^, and Mg^2+^. (b) Molar ratios based on the zero-shear viscosity peaks observed in Figure 2 (2.0, 3.1, and 3.5 for Na^+^, Ca^2+^, and Mg^2+^, respectively). The dashed lines on the graph denote the concentrations at which turbidity begins.

To investigate the influence of selected cation molar concentrations on the zero-shear viscosity of CAPB/SDBS solutions, surfactant mixtures were prepared with a constant molar ratio of 2.0 for all types of ions (Figure 3a). Additionally, mixtures were analyzed at the molar ratios corresponding to the maximum zero-shear viscosity observed in Figure 2: 2.0 for Na^+^ ions, 3.1 for Ca^2+^ ions, and 3.5 for Mg^2+^ ions, Figure 3b (viscosity curves Figures S3 and S4 in Supporting Information).

As the molar concentration of the ions increases, the viscosity initially rises and then sharply decreases after reaching a maximum. According to the data presented in Figure 3, each cation produced a viscosity peak, although the magnitudes of these peaks varied among the different cations. Sodium ions produced the highest zero-shear viscosity, while magnesium ions resulted in the lowest. Moreover, the concentration at which the viscosity peak occurs increases in the order Na^+^ > Ca^2+^ > Mg^2+^ (Figure 3a) and in the reverse order for the systems with varying CAPB/SDBS molar ratio (Figure 3b). Observations of CAPB/SDBS solution samples indicate that the solutions remained clear immediately following the viscosity peak. However, above a certain concentration of cations, all solutions became turbid. The dashed lines on the graph denote the concentrations at which turbidity begins.

Results of zero-shear viscosity measurements presented in Figure 3b were also used to select solutions for further studies. Due to their relatively low viscosity, CAPB/SDBS solutions containing Mg^2+^ ions were chosen for Cryo-EM, SAXS, and CaBER analysis.

### 3.2 Small Amplitude Oscillatory Shear Flow

**Figure 4:**
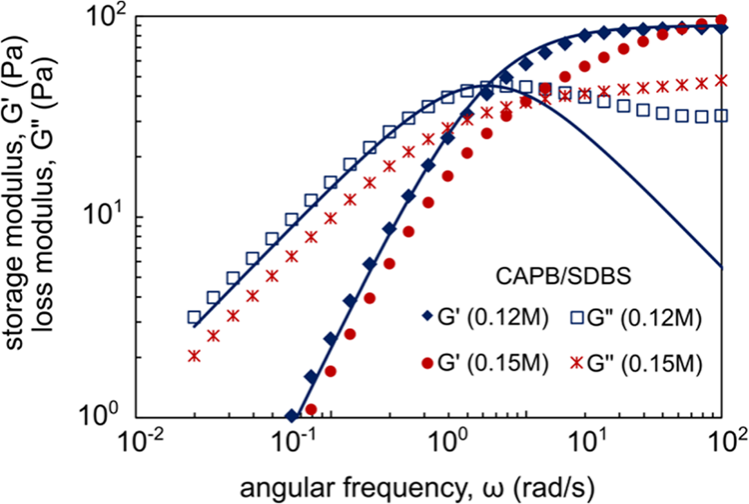
Storage modulus *G*′ and loss modulus *G*″ vs angular frequency for CAPB/SDBS solutions with molar ratio 3.5 and Mg^2+^ concentration 0.12 M and 0.15 M. Solid lines correspond to the Maxwell model fitted to the experimental data.

Small amplitude oscillatory shear measurements were performed for selected CAPB/SDBS samples with Mg^2+^ ions (the linear viscoelastic regime γ_0_ = 0.1%). Figure 4 illustrates mechanical spectra of CAPB/SDBS solutions with Mg^2+^ ions at concentrations 0.12 and 0.15 M. These concentrations correspond to samples with comparable zero-shear viscosities and, as indicated in Figure 3b, represent samples before and after the maximum zero-shear viscosity. The curves *G*′ = f(ω) i *G*″ = f(ω) obtained for the solution containing 0.12 M Mg^2+^ ions can be described using single relaxation-time Maxwell model
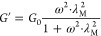
2
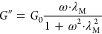
3where: *G*′ is the storage modulus, *G*″ is the loss modulus, *G*_0_ is the plateau storage modulus, and λ_M_ is the relaxation time. The relaxation time λ_M_ is calculated as the reciprocal of the angular frequency ω at which modulus *G*′ equals *G*″. By knowing λ_M_ and *G*_0_, the zero-shear viscosity can be calculated based on the equation
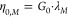
4For the sample containing 0.12 M Mg^2+^ ions, plateau modulus was determined to be *G*_0_ = 87.1 Pa, with relaxation time of λ_M_ = 0.147 s, and a zero-shear viscosity calculated using eq 4 as η_0,M_ = 12.8 Pa·s. This value is in close agreement with the zero-shear viscosity obtained under shear flow conditions (10.12 Pa·s). In the literature, Maxwellian behavior characterized by a single relaxation time is typically regarded as strong evidence for the presence of worm-like micelles. However, there are now reports of fluids whose mechanical spectra can be accurately described by the Maxwell model, despite the absence of classic worm-like micelles.^50,51^ The single relaxation-time Maxwell model was found to be inadequate for describing the mechanical spectrum of the CAPB/SDBS solution containing 0.15 M Mg^2+^ ions. For this solution, the relaxation time, estimated from the intersection of the *G*′ = f(ω) and *G*″ = f(ω) curves, was shorter, with value λ_M_ = 0.100 s.

Data presented in Figure 4 further indicate that the values of the *G*′ and *G*″ moduli for the 0.15 M Mg^2+^ sample increase across the entire angular frequency range. Consequently, the plateau modulus *G*_0_ was determined based on the dependence of the loss tangent (tan δ) on oscillation frequency ω. Specifically, *G*_0_ was taken as the *G*′ value corresponding to the minimum tan δ, in accordance with the method proposed by Wu.^52^ Using this approach, the estimated plateau modulus was *G*_0_ = 95.7 Pa, while the zero-shear viscosity calculated using eq 4 was η_0,M_ = 9.61 Pa·s, which is also consistent with the shear flow measurements (9.95 Pa·s). The oscillatory rheology results suggest that, despite having similar zero-shear viscosities, the solutions containing 0.12 and 0.15 M Mg^2+^ ions exhibit distinct differences in micellar microstructure.

### 3.3 Extensional Flow

To evaluate the rheological properties in extensional flow and their relationship with salt concentration, capillary thinning measurements were conducted on CAPB/SDBS samples (molar ratio 3.5) with magnesium ions at concentrations ranging from 0.12 to 0.2 M, both before and after the viscosity peak. The solutions with magnesium ion concentrations of 0.12 and 0.15 M exhibited similar zero-shear viscosities of 10.12 and 9.95 Pa·s, respectively. In addition to viscous, elastic, and capillary forces, inertia and gravity can also have a significant impact on capillary thinning. According to Clasen et al.,^53,54^ the effects of inertia and gravity on capillary thinning are negligible when the Ohnesorge number exceeds 1.
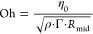
5and the Bond number is much less than 0.2.
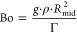
6where ρ is the density, Γ is the surface tension (due to the high viscosity of CAPB/SDBS solutions with the addition of Mg^2+^, the calculations utilized the surface tension value measured for the solution without added salt, Γ = 0.035 N/m), *g* is the gravitational constant, and *R*_mid_ is midfilament radius. For the CAPB/SDBS solutions used in the study, the estimated minimum Oh number was 5.89, and the maximum Bo number was 0.005. Therefore, it can be concluded that the effects of inertia and gravity on capillary thinning were minimal.

**Figure 5:**
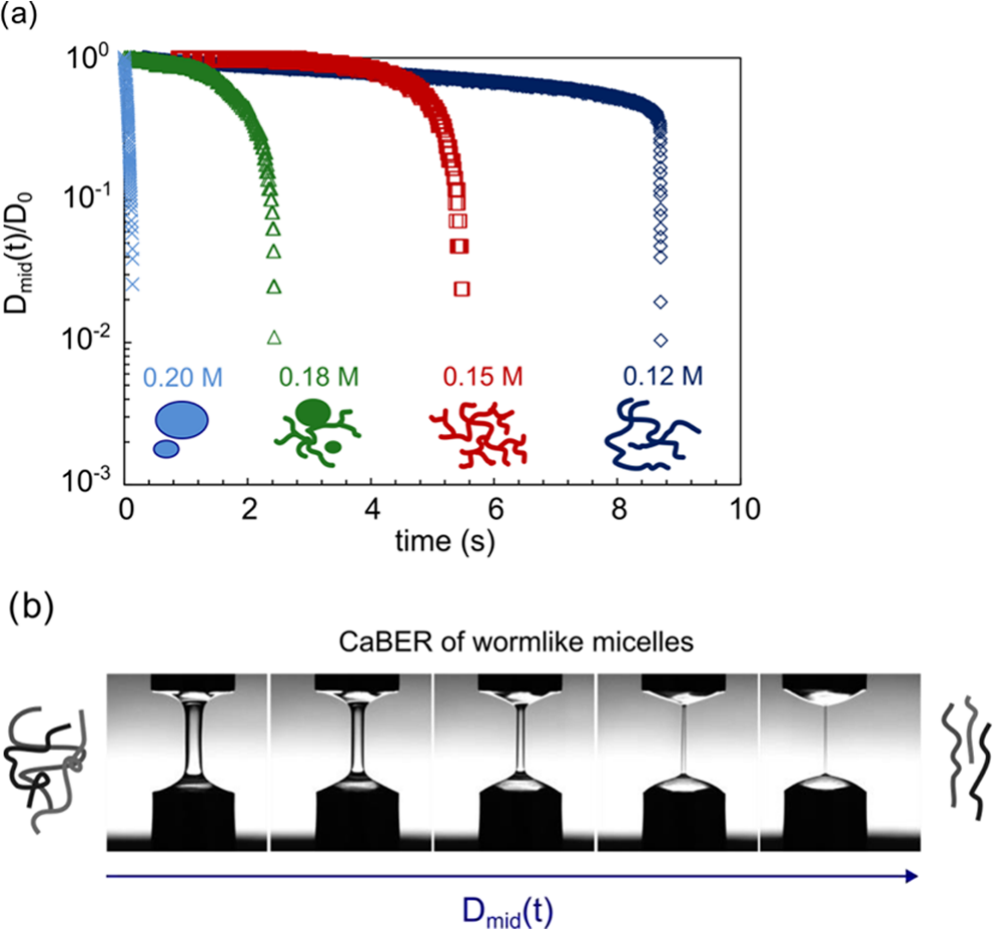
(a) Midfilament diameter as a function of time for CAPB/SDBS solutions containing different amounts of Mg^2+^ ions. The values of zero-shear viscosity were, respectively: 10.12 Pa·s for the sample with 0.12 M Mg^2+^, 9.95 Pa·s (0.15 M), 2.81 Pa·s (0.18 M), 1.16 Pa·s (0.20 M). (b) Images of the filament thinning, i.e., evolution of *D*_mid_(*t*) during the extensional rheometry experiment.

The data presented in Figure 5 reveal that the longest capillary breakup time τ_b_ (8,71 s) was observed at a magnesium ion concentration of 0.12 M. Increasing the Mg^2+^ concentration to 0.15 M decreased the capillary breakup time to about 5.55 s. Further increments in magnesium ion concentration to 0.18 and 0.2 M resulted in a dramatic reduction in breakup time to approximately 2.5 and 0.17 s, respectively. Although solutions with 0.12 and 0.15 M magnesium ions exhibited similar zero-shear viscosity, significant differences were observed in extensional flow and small amplitude oscillatory shear flow (λ_M_ = 0.147 s for CAPB/SDBS system with 0.12 M Mg^2+^, and λ_M_ = 0.100 s for system with 0.15 M Mg^2+^). These findings indicate a substantial change in the micellar microstructure, which strongly influences the fluid flow under uniaxial stretching conditions. Earlier, Chellamuthu and Rothstein^55^ and also Fischer et al.^56^ noticed significant differences between the extensional flow of entangled worm-like micelles and branched worm-like micelles.

### 3.4 Cryogenic Electron Microscopy (Cryo-EM)

**Figure 6:**
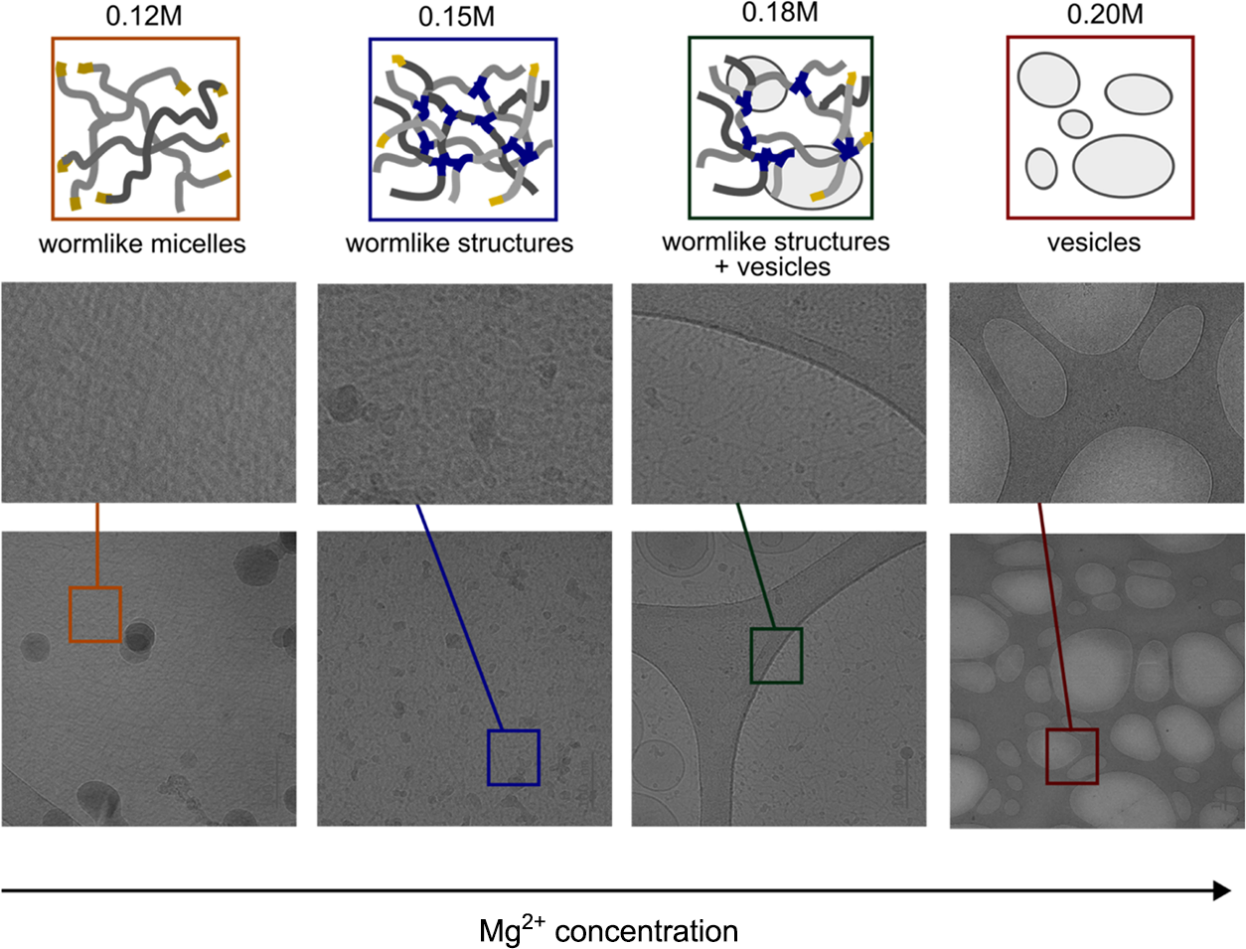
Cryo-EM images of CAPB/SDBS = 3.5 mixtures at increasing concentrations of Mg^2+^ ions. The first row presents a schematic representation of the microstructure based on the images with ion concentration values.

The micellar microstructure changes as the concentration of cations in CAPB/SDBS solutions rises as shown by rheological measurements in both shear and extensional flow. To elucidate these changes in micellar shape, Cryo-EM images were captured for CAPB/SDBS solutions with Mg^2+^ ions. The images and corresponding micellar microstructure schematics are presented in Figure 6. For samples with Mg^2+^ concentrations of 0.12 and 0.15 M (before and after the zero-shear viscosity peak, which occurred for concentration 0.14 M), a transition from worm-like micelles to worm-like structures was observed. As previously noted, these solutions exhibited similar zero-shear viscosities of approximately 10 Pa·s. At a magnesium ion concentration of 0.18 M, turbidity was first observed, and both branched worm-like micelles (worm-like structures) and vesicle micelles were present. At a concentration of 0.2 M magnesium ions, only vesicle micelles were observed. These changes in micellar shape explain the viscosity peak observed with increasing salt concentration, and they are qualitatively consistent with previously published Cryo-EM results.

### 3.5 Small-Angle X-ray Scattering (SAXS)

**Figure 7:**
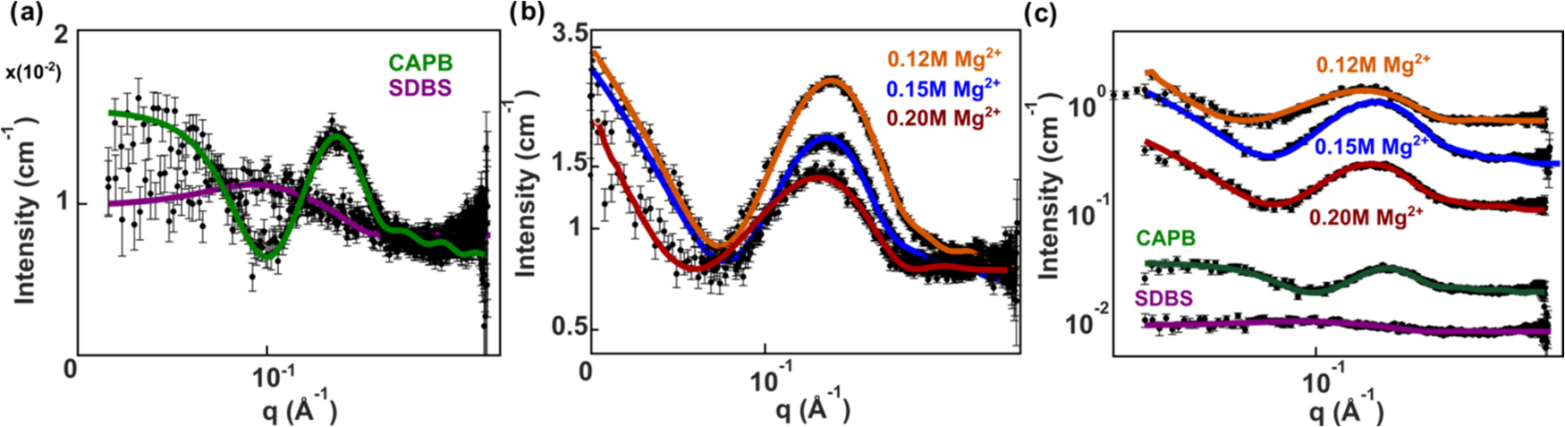
Small-angle X-ray scattering data (black circles) and model fits (solid colored lines). (a) Pure CAPB (fit with core–shell sphere model) and pure SDBS (fit with core–shell ellipsoid model). (b) Mixtures of CAPB/SDBS (77:23) with increasing Mg^2+^ concentration (0.12 and 0.15 M: fit with core–shell cylinder; 0.20 M: fit with core–multishell/vesicle model). (c) Scattering data from Figure 6a,b are compared and shifted by a factor of 2.5 for better illustration.

SAXS experiments were performed to investigate the micellar structure near the viscosity peak for CAPB/SDBS systems with increasing Mg^2+^ ion concentrations. The samples were chosen near the viscosity peak for a given salt curve (Figure 7). All studied samples have a similar profile in the high *q*-region with a broad peak, which is typical for core–shell scattering of micellar radial cross sections.^57^ The minimum before the peak is correlated with the radius of the objects^58,59^ and the shift to a smaller *q* in the presence of electrolytes is an indication of micellar growth. The main difference in the scattering pattern is in the low *q*-region, which corresponds to bigger objects, following the relation *d* = 2π*/q*.

A core–shell cylinder model^60,61^ accurately describes a similar surfactant SLES/CAPB^48^ system in the presence of salts, making it suitable for X-ray scattering of micellar systems. While rheological measurements of CAPB/SDBS are prevalent in the literature,^62,63^ the scattering of these systems is absent. The scattering and model fits of pure CAPB and SDBS are presented in Figure 7a, as well as the scattering and model fits of CAPB/SDBS molar ratio 3.5 (78:22 mol %) with increasing Mg^2+^ concentration in Figure 6b. To account for concentration, CAPB was fitted to a core–shell sphere model with a hard sphere structure factor. The fits resulted in a total core–shell radius of 27.3 Å, similar to recent SANS measurements.^63^ SDBS was fit to a core–shell ellipsoid structure with a Hayter Penfold mean spherical approximation structure factor to account for the charge-based structure factor. For simplicity, mixed CAPB/SDBS/MgCl_2_ systems were modeled as core–shell elliptical cylinders. With the available *q*-range for SAXS measurements, a flexible cylinder-type model will not yield additional information for these systems.

The mixtures in the presence of increasing MgCl_2_ concentration are analyzed with model fitting of core–shell structures ranging from spheres to cylinders and vesicle architectures, as indicated in (Table 1). The results for CAPB/SDBS systems with increasing concentrations of Mg^2+^ (0.15 and 0.18 M) in the higher-*q* region were fit with a core–shell elliptical cylinder model. For the 0.2 M concentration, the observed vesicles (corroborated by cryo-EM images) were fit with a core multishell sphere model. The core–multishell model enables appropriate modeling of the bilayer cross-section as observed with the X-ray scattering of vesicles. Accordingly, in the fitting parameters, the core has the scattering length density (SLD) of water, followed by three shells representing the headgroup–tailgroup–headgroup regions, with the solvent being water as well. The resultant total thickness of the bilayer is 42.21 Å. The fitting parameters are detailed in Table 1.

**Table 1:** Small-Angle X-ray Scattering Model Fitting Parameters for CAPB and SDBS Pure and Mixed Systems

system	form factor	SLD core (·10^–6^/Å^2^)	SLD shell (·10^–6^/Å^2^)	total major radius (Å)	total minor radius (Å)	χ^2^
CAPB	core–shell sphere	9.74	9.43 ± 0.0021	27.3 ± 0.0134		1.64
SDBS	core–shell ellipsoid	9.74	8.78 ± 0.0028	25.8 ± 0.5581	18.1 ± 0.4218	1.70

200 mM CAPB/SDBS (77:23) with Mg^2+^							
system	form factor	SLD core (·10^–6^/Å^2^)	SLD shell (·10^–6^/Å^2^)	length (Å)	total major radius (Å)	total minor radius (Å)	χ^2^
0.12 M	core–shell ell. cylinder	9.74	8.93	>628	23.9	17.2	2.53
0.15 M	core–shell ell. cylinder	9.76	9.04	>628	22.7	16.6	2.55

system	form factor	SLD core (·10^–6^/Å^2^)	SLD shell 1, 3 (·10^–6^/Å^2^)	SLD shell 2 (·10^–6^/Å^2^)	total major radius (Å)	bilayer thickness (Å)	χ^2^
0.20 M	core multishell sphere	9.47	9.1 ± 0.0035	9.74 ± 0.0016	>628	42.2	1.65

### 3.6 Discussion

Cryo-EM images and SAXS data indicate that the occurrence of zero-shear viscosity peaks with increasing concentrations of metal cations in CAPB/SDBS solutions is due to the transformation of entangled worm-like micelles into branched worm-like micelles.^15^ According to the analysis by Lequeux,^64^ the formation of branches facilitates the diffusion of micellar chains by allowing them to slide along the micellar contour, thereby acting as points for stress relaxation. At higher salt concentrations (Figure 3), vesicles are formed accompanied by the turbidity of the solutions. The transformation from entangled worm-like micelles to branched worm-like micelles, and subsequently to vesicles can be explained based on the packing theory proposed by Israelachvili.^26^

As the concentration of cations increases, screening of the negatively charged COO^–^ and SO_3_^–^ groups occurs, resulting in a decrease in the effective headgroup area and an increase in the packing parameter *p*. Our studies reveal that the molar ratio of CAPB/SDBS, and the cation concentration at which the zero-shear viscosity peak is observed, vary depending on the type of cation introduced into the solution. Since we used both monovalent and divalent cations (counterions), it was predictable that differences would arise due to varying valency. However, the key question is what happens when both counterions are same valency, but they differ in hydrated ion radius and *Z*/*R* ratio. In our studies, the increase in the molar ratio of CAPB/SDBS at which the zero-shear viscosity peak is observed, following the addition of Na^+^, Ca^2+^, and Mg^2+^ ions, corresponds to the ability of these metal cations to form close pairs with the anionic surfactant headgroups.^48,65^ Among the cations used in the measurements, Mg^2+^ interacts most strongly with the −COO^–^ group, whereas Na^+^ interacts the least. Conversely, for the −SO_3_^–^ group, the interaction is opposite. Specifically, the weakest interactions occur between sulfonate headgroups and magnesium ions, while the strongest interactions are between sulfonate headgroups and Na^+^ ions.^48,65^ The screening of −COO^–^ and −SO_3_^–^ groups by the addition of cations to the solution reduces the repulsion between surfactant headgroups, thereby increasing the packing parameter *p*. As a result, worm-like micelles (WLM) elongate or transform into bilayer structures. Since magnesium ions have a greater affinity for −COO^–^ groups than for −SO_3_^–^ groups, more effective screening and a corresponding increase in the packing parameter *p* will occur when the surfactant mixture has a relatively high SDBS content. For sodium ions, which have a greater affinity for the SO^3–^ group, the situation is the opposite: the packing parameter *p* will increase with a relatively higher content of COO^–^ ions. Consequently, in solutions containing calcium ions, the zero-shear viscosity peak will occur at intermediate molar ratios of CAPB to SDBS. This mechanism aligns with the one proposed by Mitrinova et al.^48^ for SLES/CAPB solutions containing different cations. However, there are differences between the published results for SLES/CAPB solutions and the findings presented in this study for CAPB/SDBS solutions, particularly concerning the cation concentration at which the zero-shear viscosity peak occurs. For SLES/CAPB solutions (molar ratio 0.5), the cation concentration at which the maximum zero-shear viscosity was observed increased in the order of Na^+^ > Mg^2+^ > Ca^2+^. In contrast, in our studies, for CAPB/SDBS solutions at the same molar ratio (CAPB/SDBS = 2, equivalent to SDBS/CAPB = 0.5), the order was Na^+^ > Ca^2+^ > Mg^2+^ (Figure 3a). Mitrinova et al.^48^ correlated the cation concentration at which the viscosity peak occurs for SLES/CAPB solutions with the ratio between ion charge *Z* and hydrated radius *R* (the *Z*/*R* ratio for sodium, calcium, and magnesium ions is 2.8, 4.9, and 4.7, respectively). A higher *Z*/*R* ratio indicates greater interaction energy between the surfactant anion and the counterion, resulting in a lower electrolyte concentration needed to achieve a packing parameter corresponding to WLM formation.^48^ While this explanation is reasonable, it may not always be decisive, as other factors also contribute to how cations, even those with the same valency, influence micellar formation.

Notably, in CAPB/SDBS solutions, the relationship between the concentration of calcium and magnesium ions and the *Z*/*R* ratio does not apply. We propose two possible reasons for the maximum viscosity occurring at a lower concentration of magnesium ions compared to calcium ions. The first reason could be the higher affinity of Mg^2+^ ions for the −COO^–^ group compared to Ca^2+^ ions, leading to stronger screening of the carboxyl groups in the CAPB molecule and indicating an increasing SLD shell from SAXS (details in Table 1). This stronger screening reduces the effective surface area more significantly, resulting in an increased packing parameter. The second reason could be related to the partial dehydration of −SO_3_^–^ groups or amidopropyl linkers. Evidence for the partial dehydration of these groups with increasing magnesium ion concentration is suggested by the reduction in micelle diameter as determined by SAXS measurements (Table 1). Recent studies by Williams et al.^63^ on sodium lauryl *n*-(ethylene glycol) sulfate solutions with added NaCl indicate that the addition of salt not only screens the SO_3_^–^ groups but also induces partial dehydration of the ether linkers in the micelles. To be sure, a further study would be required with contrast variation SANS.

The data presented in Figure 3 indicate that the maximum zero-shear viscosity values decrease with the addition of specific cations in the order of Na^+^ > Ca^2+^ > Mg^2+^. A similar trend was reported by Mitrinova et al.^48^ for SELS/CAPB solutions. The observed order of changes in the zero-shear viscosity peak and maximum values corresponds to the Hofmeister series.^38,44,45,66^ For CAPB/SDBS solutions, there is a correlation between the hydrated cation diameter (0.358 nm for Na^+^, 0.412 nm for Ca^2+^, and 0.428 nm for Mg^2+^) and both the maximum zero-shear viscosity value and the salt concentration at which the zero-shear viscosity peak occurs. Magnesium ions, which are kosmotropes, bind the most water molecules, followed by calcium ions, and sodium ions, which are chaotropes, bind the least. Consequently, the greatest dehydration of SO^3–^ groups or amidopropyl linkers is induced by Mg^2+^ ions and the least by Na^+^ ions. Greater dehydration of surfactant headgroups, combined with charge screening, can cause a significant reduction in the effective surface area occupied by the polar head on the micelle surface, thereby increasing the packing parameter. With the addition of magnesium ions, the maximum zero-shear viscosity peak value is the lowest, followed by the highest with the addition of sodium ions. Figure 3b also shows the connection between zero-shear viscosity and cation concentration for the CAPB and SDBS molar ratios where the zero-shear viscosity peak was found. In this case, the cation concentration at which the zero-shear viscosity peak appears increases in the order of Na^+^ < Ca^2+^ < Mg^2+^. This order of cation concentrations correlates with the increasing number of −COO^–^ groups in the solution, for which magnesium ions have the highest affinity and also with valency, as it was explained before. However, the changes in the viscosity maxima follow the same pattern as in Figure 2.

## 4 Conclusions

This study presents findings on the impact of Ca^2+^, Mg^2+^, Na^+^ cations on the rheological properties of cocamidopropyl betaine (CAPB) and sodium dodecylbenzenesulfonate (SDBS) surfactant systems under shear and extensional flow. Our research demonstrates that the maximum zero-shear viscosity as a function of salt concentration (salt curve) of CAPB/SDBS mixtures is highly dependent on the type of cation added and the molar ratio of the surfactants. We found that the concentration of Ca^2+^ cations at which the zero-shear viscosity peak occurs is higher than that for Mg^2+^ cations, even though they are both divalent cations. Cryo-EM imaging and SAXS measurements reveal that the observed changes in zero-shear viscosity and extensional viscosity with increasing cation concentration are due to the transformation of entangled worm-like micelles into a branched network and eventually into vesicles at higher cation concentrations (applicable to all types of cations). Two key factors explain the differences in the effects of Ca^2+^, Mg^2+^ and Na^+^ on worm-like micelle formation. The first is the distinct affinity of the studied cations for the −COO^–^ and SO^3–^ groups. The second factor may involve the partial dehydration of the SO^3–^ groups or the amidopropyl linkers. Variations in dehydration and the screening of the surfactant polar heads induced by sodium, calcium, and magnesium cations likely result in differential reductions in the effective surface area occupied by the polar heads on the micelle surface. This, in turn, differently influences the shape of the formed micelles and, consequently, the rheological properties of the CAPB/SDBS solutions. The observed order and magnitude of changes in the zero-shear viscosity peak for different ions align with, and can be explained by, the Hofmeister series.

## Supporting Information Available

Additional viscosity curves for the measured systems, offering deeper insights into the data (PDF)

## References

[ref1] Shaban S. M.; Kang J.; Kim D. H. Surfactants: recent advances and their applications. Compos. Commun. 2020, 22, 100537 10.1016/j.coco.2020.100537.

[ref2] Farias C. B. B.; Almeida F. C.; Silva I. A.; Souza T. C.; Meira H. M.; Rita de Cássia F.; Sarubbo L. A.; et al. Production of green surfactants: Market prospects. Electron. J. Biotechnol. 2021, 51, 28–39. 10.1016/j.ejbt.2021.02.002.

[ref3] Badmus S. O.; Amusa H. K.; Oyehan T. A.; Saleh T. A. Environmental risks and toxicity of surfactants: an overview of analysis, assessment, and remediation techniques. Environ. Sci. Pollut. Res. 2021, 28, 62085–62104. 10.1007/s11356-021-16483-w. PMC8480275 34590224

[ref4] Dreiss C. A. Wormlike micelles: where do we stand? Recent developments, linear rheology and scattering techniques. Soft Matter 2007, 3 (8), 956–970. 10.1039/b705775j. 32900044

[ref5] Berret J. F. Rheology of Wormlike Micelles: Equilibrium Properties and Shear Banding Transitions. Mol. Gels 2006, 19, 667–720. 10.1007/1-4020-3689-2_20.

[ref6] Wang J.; Liu D.; Huang Z.; Zheng C. CO_2_ responsive wormlike micelles based on sodium oleate, potassium chloride and N, N-dimethylethanolamine. J. Dispersion Sci. Technol. 2018, 39 (11), 1606–1612. 10.1080/01932691.2018.1452758.

[ref7] Różańska S.; Warmbier E.; Wagner P.; Różański J. Structural changes of viscoelastic solutions of zwitterionic andanionic surfactant mixtures under the influence of simple salt. Chem. Process Eng.: New Fron. 2023, 44 (2), e10 10.24425/cpe.2023.144696.

[ref8] Parker A.; Fieber W. Viscoelasticity of anionic wormlike micelles: effects of ionic strength and small hydrophobic molecules. Soft Matter 2013, 9, 1203–1213. 10.1039/C2SM27078A.

[ref9] Fieber W.; Scheklaukov A.; Kunz W.; Pleines M.; Benczédi D.; Zemb T. Towards a general understanding of the effects of hydrophobic additives on the viscosity of surfactant solutions. J. Mol. Liq. 2021, 329, 115523 10.1016/j.molliq.2021.115523.

[ref10] Helgeson M. E.; Vasquez P. A.; Kaler E. W.; Wagner N. J. Rheology and spatially resolved structure of cetyltrimethylammonium bromide wormlike micelles through the shear banding transition. J. Rheol. 2009, 53 (3), 727–756. 10.1122/1.3089579.

[ref11] Lin Z.; Cai J. J.; Scriven L. E.; Davis H. T. Spherical-to-wormlike micelle transition in CTAB solutions. J. Phys. Chem. A 1994, 98 (23), 5984–5993. 10.1021/j100074a027.

[ref12] Sylwia R.; Rózański J.; Wagner P.; Warmbier E. Pressure drops during the flow of solutions of cocamidopropyl betaine and cocamide DEA mixtures with the addition of ethylene glycol. Pol. J. Chem. Technol. 2022, 24 (4), 67–71. 10.2478/pjct-2022-0030.

[ref13] Pandya N.; Rajput G.; Janni D. S.; Subramanyam G.; Ray D.; Aswal V.; Varade D. SLES/CMEA mixed surfactant system: Effect of electrolyte on interfacial behavior and microstructures in aqueous media. J. Mol. Liq. 2021, 325, 115096 10.1016/j.molliq.2020.115096.

[ref14] Schubert B. A.; Kaler E. W.; Wagner N. J. The microstructure and rheology of mixed cationic/anionic wormlike micelles. Langmuir 2003, 19, 4079–4089. 10.1021/la020821c.

[ref15] Różańska S. Rheology of wormlike micelles in mixed solutions of cocoamidopropyl betaine and sodium dodecylbenzenesulfonate. Colloids Surf., A 2015, 482, 394–402. 10.1016/j.colsurfa.2015.06.045.

[ref16] Różańska S.; Różański J. Shear and extensional rheology of aqueous solutions of cocamidopropyl betaine and sodium dodecyl sulfate mixture. J. Dispersion Sci. Technol. 2020, 41, 733–741. 10.1080/01932691.2019.1611442.

[ref17] Jamadagni S. N.; Ko X.; Thomas J. B.; Eike D. M. Salt and pH-dependent viscosity of SDS/LAPB solutions: experiments and a semiempirical thermodynamic model. Langmuir 2021, 37, 8714–8725. 10.1021/acs.langmuir.1c00964. 34270265

[ref18] López-Díaz D.; Castillo R. The wormlike micellar solution made of a zwitterionic surfactant (TDPS), an anionic surfactant (SDS), and brine in the semidilute regime. J. Phys. Chem. B 2010, 114, 8917–8925. 10.1021/jp102108y. 20572649

[ref19] Qiao Y.; Lin Y.; Wang Y.; Li Z.; Huang J. Metal-driven viscoelastic wormlike micelle in anionic/zwitterionic surfactant systems and template-directed synthesis of dendritic silver nanostructures. Langmuir 2011, 27 (5), 1718–1723. 10.1021/la104447d. 21218845

[ref20] Naskar B.; Mondal S.; Moulik S. P. Amphiphilic activities of anionic sodium cholate (NaC), zwitterionic 3-[(3-cholamidopropyl) dimethylammonio]-1-propanesulfonate (CHAPS) and their mixtures: A comparative study. Colloids Surf., B 2013, 112, 155–164. 10.1016/j.colsurfb.2013.07.029. 23973673

[ref21] Maswal M.; Dar A. A. Mixed micelles of sodium cholate and Brij30: Their rheological behaviour and capability towards solubilization and stabilization of rifampicin. Colloids Surf., A 2013, 436, 704–713. 10.1016/j.colsurfa.2013.07.039.

[ref22] Hu Y.; Chen Y.; Cai Z.; Jin X.; Fan L.; Han J.; Guo R. Brij 30 induced transition of rodlike micelles to wormlike micelles and gels in the imidazole ionic liquid surfactants: the alkyl chain length effect. Langmuir 2022, 38 (10), 3051–3063. 10.1021/acs.langmuir.1c02602. 35226483

[ref23] Hu Y.; Han J.; Guo R. Wormlike micelle to gel transition induced by brij 30 in ionic liquid-type surfactant aqueous solution. Wuli Huaxue Xuebao 2020, 36, 1909049 10.3866/PKU.WHXB201909049.

[ref24] Kunieda H.; Rodriguez C.; Tanaka Y.; Kabir M. H.; Ishitobi M. Effects of added nonionic surfactant and inorganic salt on the rheology of sugar surfactant and CTAB aqueous solutions. Colloids Surf., B 2004, 38 (3–4), 127–130. 10.1016/j.colsurfb.2004.01.014. 15542313

[ref25] Israelachvili J. N.; Mitchell D. J.; Ninham B. W. Theory of self-assembly of hydrocarbon amphiphiles into micelles and bilayers. J. Chem. Soc., Faraday Trans. 2 1976, 72, 1525–1568. 10.1039/F29767201525.

[ref26] Israelachvili J. Self-assembly in two dimensions: surface micelles and domain formation in monolayers. Langmuir 1994, 10 (10), 3774–3781. 10.1021/la00022a062.

[ref27] Lutz-Bueno V.; Isabettini S.; Walker F.; Kuster S.; Liebi M.; Fischer P. Ionic micelles and aromatic additives: a closer look at the molecular packing parameter. Phys. Chem. Chem. Phys. 2017, 19 (32), 21869–21877. 10.1039/C7CP03891G. 28787055

[ref28] Isabettini S.; Boni L. J.; Baumgartner M.; Saito K.; Kuster S.; Fischer P.; Lutz-Bueno V. Higher salt hydrophobicity lengthens ionic wormlike micelles and stabilizes them upon heating. Langmuir 2021, 37 (1), 132–138. 10.1021/acs.langmuir.0c02608. 33356307

[ref29] Fischer P.; Lutz-Bueno V. Glycyrrhizic acid aggregates seen from a synthetic surfactant perspective. Phys. Chem. Chem. Phys. 2024, 26 (4), 2806–2814. 10.1039/D3CP04835G. 38196347 PMC10806618

[ref30] Clendennen S. K.; Boaz N. W. Betaine amphoteric surfactants—synthesis, properties, and applications. Biobased Surfactants (2nd Ed.) 2019, 2, 447–469. 10.1016/B978-0-12-812705-6.00014-9.

[ref31] Floyd D. 11 Applications of Amphoterics-Based Formulations Handbook of Detergents, Part E: Applications 2008; Vol. 20084959, pp 287–299.

[ref32] Różański J.; Różańska S.; Mitkowski P. T.; Szaferski W.; Wagner P.; Frankiewicz A. Drag reduction in the flow of aqueous solutions of a mixture of cocamidopropyl betaine and cocamide DEA. Energies 2021, 14 (9), 2683 10.3390/en14092683.

[ref33] Han W.; Tan J.; Peng L.; Liu L.; Zhou X.; Zhang W.; Shi B. Ecotoxicity and micellization behavior of anionic surfactant sodium dodecylbenzene sulfonate (SDBS) and its mixtures with nonionic surfactant fatty alcohol-polyoxyethylene ether (AEO). Aquat. Toxicol. 2019, 216, 105313 10.1016/j.aquatox.2019.105313. 31568897

[ref34] Su Y.; Xie Q.; Chen C.; Zhang Q.; Ma M.; Yao S. Electrochemical quartz crystal microbalance studies on enzymatic specific activity and direct electrochemistry of immobilized glucose oxidase in the presence of sodium dodecyl benzene sulfonate and multiwalled carbon nanotubes. Biotechnol. Prog. 2008, 24 (1), 262–272. 10.1021/bp070256+. 18062696

[ref35] Sun Z.; Ji Y.; Wang H.; Zhang J.; Yuan C.; Kang M.; Feng Y.; Yin H. Impact of hydroxyethyl headgroup on long-chain quaternary ammonium cationic surfactants: Solubility, surface activities, self-assembly behaviors, and rheological properties. Colloids Surf., A 2024, 700, 134831 10.1016/j.colsurfa.2024.134831.

[ref36] Chu Z.; Dreiss C. A.; Feng Y. Smart wormlike micelles. Chem. Soc. Rev. 2013, 42 (17), 7174–7203. 10.1039/c3cs35490c. 23545844

[ref37] Croce V.; Cosgrove T.; Dreiss C. A.; Maitland G.; Hughes T.; Karlsson G. Impacting the length of wormlike micelles using mixed surfactant systems. Langmuir 2004, 20 (19), 7984–7990. 10.1021/la0487500. 15350062

[ref38] Mütze A.; Heunemann P.; Fischer P. On the appearance of vorticity and gradient shear bands in wormlike micellar solutions of different CPCl/salt systems. J. Rheol. 2014, 58 (6), 1647–1672. 10.1122/1.4887536.

[ref39] Joshi J.; Aswal V.; Goyal P. Effect of sodium salicylate on the structure of micelles of different hydrocarbon chain lengths. Physica B: Condens. Matter 2007, 391 (1), 65–71. 10.1016/j.physb.2006.08.050.

[ref40] Abdel-Rahem R. A.; Reger M.; Hloucha M.; Hoffmann H. Rheology of aqueous solutions containing SLES, CAPB, and microemulsion: influence of cosurfactant and salt. J. Dispersion Sci. Technol. 2014, 35, 64–75. 10.1080/01932691.2013.774282.

[ref41] Tang X.; Zou W.; Koenig P. H.; McConaughy S. D.; Weaver M. R.; Eike D. M.; Schmidt M.; Larson R. G. Multiscale modeling of the effects of salt and perfume raw materials on the rheological properties of commercial threadlike micellar solutions. J. Phys. Chem. B 2017, 121 (11), 2468–2485. 10.1021/acs.jpcb.7b00257. 28225285

[ref42] Abezgauz L.; Kuperkar K.; Hassan P. A.; Ramon O.; Bahadur P.; Danino D. Effect of Hofmeister anions on micellization and micellar growth of the surfactant cetylpyridinium chloride. J. Colloid Interface Sci. 2010, 342, 83–92. 10.1016/j.jcis.2009.08.045. 19939405

[ref43] Oelschlaeger C.; Suwita P.; Willenbacher N. Effect of counterion binding efficiency on structure and dynamics of wormlike micelles. Langmuir 2010, 26 (10), 7045–7053. 10.1021/la9043705. 20180526

[ref44] Hofmeister F. 24. Zur Lehre von der Wirkung der Salze. Arch. Exp. Pathol. Pharmakol. 1890, 27, 395–413. 10.1007/BF01834645.

[ref45] Hofmeister F. Zur Lehre von der Wirkung der Salze. Arch. Exp. Pathol. Pharmakol. 1891, 28, 210–238. 10.1007/BF01824334.

[ref46] Collins K. D.; Neilson G. W.; Enderby J. W. Ions in water: characterizing the forces that control chemical processes and biological structure. Biophys. Chem. 2007, 128, 95–104. 10.1016/j.bpc.2007.03.009. 17418479

[ref47] Vlachy N.; Jagoda-Cwiklik B.; Vacha R.; Touraud D.; Jungwirth P.; Kunz W. Hofmeister series and specific interactions of charged headgroups with aqueous ions. Adv. Colloid Interface Sci. 2009, 146, 42–47. 10.1016/j.cis.2008.09.010. 18973869

[ref48] Mitrinova Z.; Alexandrov H.; Denkov N.; Tcholakova S. Effect of counter-ion on rheological properties of mixed surfactant solutions. Colloids Surf., A 2022, 643, 128746 10.1016/j.colsurfa.2022.128746.

[ref49] Warmbier E.; Altaee A.; Różański J.; Kazwini T.; Różańska S.; Ibrar I.; Hawari A. H.; et al. Stability of Viscoelastic Solutions: BrijL4 and Sodium Cholate Mixtures with Metal Ions Across a Broad pH and Temperature Range. Langmuir 2024, 40 (3), 1707–1716. 10.1021/acs.langmuir.3c02879. 38180900 PMC10810160

[ref50] Rincón-Londoño N.; Luviano A. S.; Tavera-Vázquez A.; Figueroa-Gerstenmaier S.; Castillo R. Synergistic effect of a fluorinated azobenzene for transforming spherical micelles into thread-like micelles with a disk-like cross-section. J. Mol. Liq. 2023, 390, 123066 10.1016/j.molliq.2023.123066.

[ref51] Luviano A. S.; Figueroa-Gerstenmaier S.; Sarmiento-Gómez E.; Rincón-Londoño N. Non-disruptive mixing of cyclodextrins and wormlike micelles in the non-dilute regime. J. Mol. Liq. 2023, 369, 120844 10.1016/j.molliq.2022.120844.

[ref52] Wu S. Chain structure and entanglement. J. Polym. Sci., Part B:Polym. Phys. 1989, 27 (4), 723–741. 10.1002/polb.1989.090270401.

[ref53] Clasen C.; Phillips P. M.; Palangetic L.; Vermant A. J. Dispensing of rheologically complex fluids: The map of misery. AIChE J. 2012, 58 (10), 3242–3255. 10.1002/aic.13704.

[ref54] Clasen C. Capillary Breakup Extensional Rheometry of Semi-Dilute Polymer Solutions. Korea-Aust. Rheol. J. 2010, 22, 331–338.

[ref55] Chellamuthu M.; Rothstein J. P. Distinguishing between linear and branched wormlike micelle solutions using extensional rheology measurements. J. Rheol. 2008, 52 (3), 865–884. 10.1122/1.2896120.

[ref56] Fischer P.; Fuller G. G.; Lin Z. Branched viscoelastic surfactant solutions and their response to elongational flow. Rheol. Acta 1997, 36, 632–638. 10.1007/BF00367359.

[ref57] Lutz-Bueno V.; Liebi M.; Kohlbrecher J.; Fischer P. Intermicellar interactions and the viscoelasticity of surfactant solutions: Complementary use of SANS and SAXS. Langmuir 2017, 33 (10), 2617–2627. 10.1021/acs.langmuir.6b04466. 28221812

[ref58] Kelly E. A.; Willis-Fox N.; Houston J. E.; Blayo C.; Divitini G.; Cowieson N.; Daly R.; Evans R. C. A single-component photorheological fluid with light-responsive viscosity. Nanoscale 2020, 12 (11), 6300–6306. 10.1039/C9NR10350C. 32162625

[ref59] Fielding L. A.; Lane J. A.; Derry M. J.; Mykhaylyk O. O.; Armes S. P. Thermo-responsive diblock copolymer worm gels in non-polar solvents. J. Am. Chem. Soc. 2014, 136 (15), 5790–5798. 10.1021/ja501756h. 24678949 PMC4015619

[ref60] Eguchi K.; Kaneda I.; Hiwatari Y.; Masunaga H.; Sakurai K. Salt-concentration dependence of the structure and form factors for the wormlike micelle made from a dual surfactant in aqueous solutions. J. Appl. Crystallogr. 2007, 40, s264–s268. 10.1107/S0021889807017888.

[ref61] Naruse K.; Eguchi K.; Akiba I.; Sakurai K.; Masunaga H.; Ogawa H.; Fossey J. S. Flexibility and cross-sectional structure of an anionic dual-surfactant wormlike micelle explored with small-angle X-ray scattering coupled with contrast variation technique. J. Phys. Chem. B 2009, 113 (30), 10222–10229. 10.1021/jp9019415. 19572674

[ref62] Abdel-Rahem R. A.; Al-Akayleh F.; Al-Remawi M. Tensiometric and rheological investigations of single and mixed systems consisting of cocamidopropyl betaine (CAPB) and sodium dodecyl benzene sulfonate (SDBS) in aqueous solutions. Tenside, Surfactants, Deterg. 2023, 60 (3), 214–222. 10.1515/tsd-2022-2492.

[ref63] Williams A. P.; Faber J. M.; Recsei C.; de Campo L.; Darwish T. A.; Tuck K. L.; Tabor R. F. Salt-induced linker dehydration modulates micellar structure in ethyl-linked sulfate surfactants. J. Phys. Chem. B 2024, 128 (27), 6648–6653. 10.1021/acs.jpcb.4c03429. 38935971

[ref64] Lequeux F. Reptation of connected wormlike micelles. Europhys. Lett. 1992, 19 (8), 675 10.1209/0295-5075/19/8/003.

[ref65] Sarmiento-Gomez E.; Lopez-Diaz D.; Castillo R. Microrheology and characteristic lengths in wormlike micelles made of a zwitterionic surfactant and SDS in brine. J. Phys. Chem. B 2010, 114 (38), 12193–12202. 10.1021/jp104996h. 20825212

[ref66] Assaf K. I.; Nau W. M. The chaotropic effect as an assembly motif in chemistry. Angew. Chem., Int. Ed. 2018, 57 (43), 13968–13981. 10.1002/anie.201804597. PMC6220808 29992706

