# Supplementary material for: How Do the Valency and Radii of Cations Affect the Rheological Properties of Aqueous Solutions of Zwitterionic and Anionic Surfactant Mixtures?
Source: Langmuir. 2025 Jan 29;41(5):3561–71. doi: 10.1021/acs.langmuir.4c04689 (PMC11823622; doi:10.1021/acs.langmuir.4c04689)
Supplement: Supplementary file 1 — la4c04689_si_001.pdf [file la4c04689_si_001.pdf]

SUPPORTING INFORMATION

How do the valency and radii of cations affect the rheological properties of aqueous solutions of zwitterionic and anionic surfactant mixtures?

Ewelina Warmbier-Wytykowska[1*], Ashley Peter Williams[2], Jacek Rozanski[1], Peter Fischer[3], Viviane Lutz-Bueno[2], Stephan Handschin[4], Laura Baraldi[3], Jarosław Warmbier[5], Patrycja Wagner[1], Sylwia Różańska[1]

1 Institute of Chemical Technology and Engineering, Faculty of Chemical Technology, Poznan University of Technology, ul. Berdychowo 4, PL 60-965 Poznan, Poland
2 Laboratory for Neutron Scattering and Imaging, Paul Scherrer Institut, 5232 Villigen, Switzerland
3 Institute of Food, Nutrition and Health, ETH Zürich, Schmelzbergstrasse 7, 8092, Zürich, Switzerland
4 Scientific Center for Optical and Electron Microscopy, ETH Zürich, Auguste-Piccard-Hof 1, 8093 Zürich, Switzerland
5 Faculty of Control, Robotics and Electrical Engineering, Division of Control and Robotics, Poznan University of Technology, ul. Piotrowo 3a, 60-965 Poznan, Poland

Corresponding author: ewelina.warmbier@doctorate.put.poznan.pl

Number of pages: 3
Number of figures: 4
Number of schemes: 0
Number of tables: 0

Table of Contents:

Fig. S1. Viscosity curves of CAPB/SDBS solutions for various molar ratios with the 0.1M concentration of Mg^{2+} ions…………………………………………………………...…S2
Fig. S2. Viscosity curves of CAPB/SDBS solutions with the addition of 0.1M concentration of Ca^{2+} ions…………………………………………………………………………...…S2
Fig. S3. Viscosity curves of CAPB/SDBS solutions with a constant molar ratio of 3.5 and varying Mg^{2+} molar concentration……………………………………………………………S3
Fig. S4. Viscosity curves of CAPB/SDBS solutions with a constant molar ratio of 3.5 and varying Ca^{2+} ion molar concentration……………………………………………………...S3

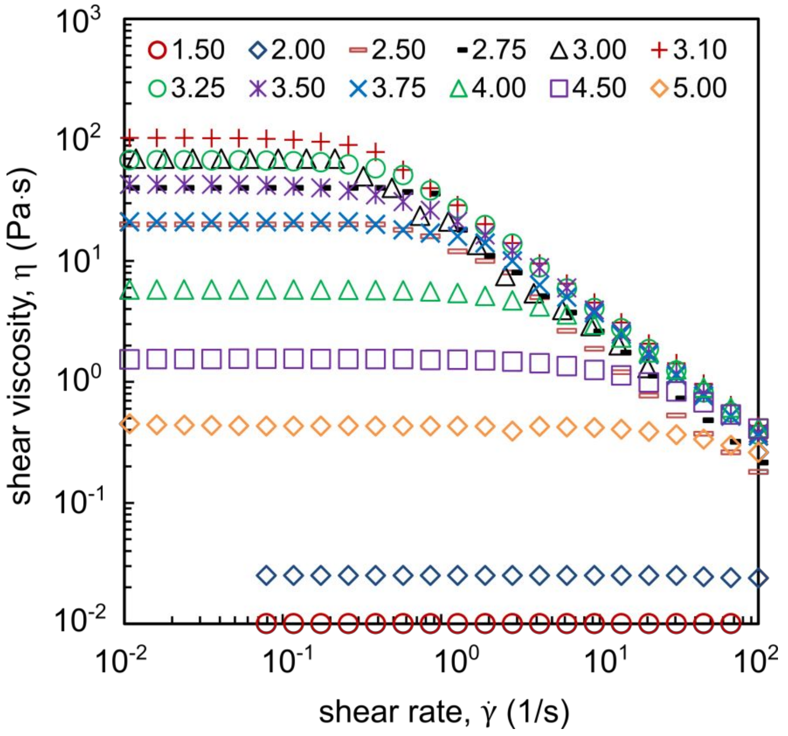

Fig. S1. Viscosity curves of CAPB/SDBS solutions for various molar ratios with the 0.1M concentration of Mg^{2+} ions

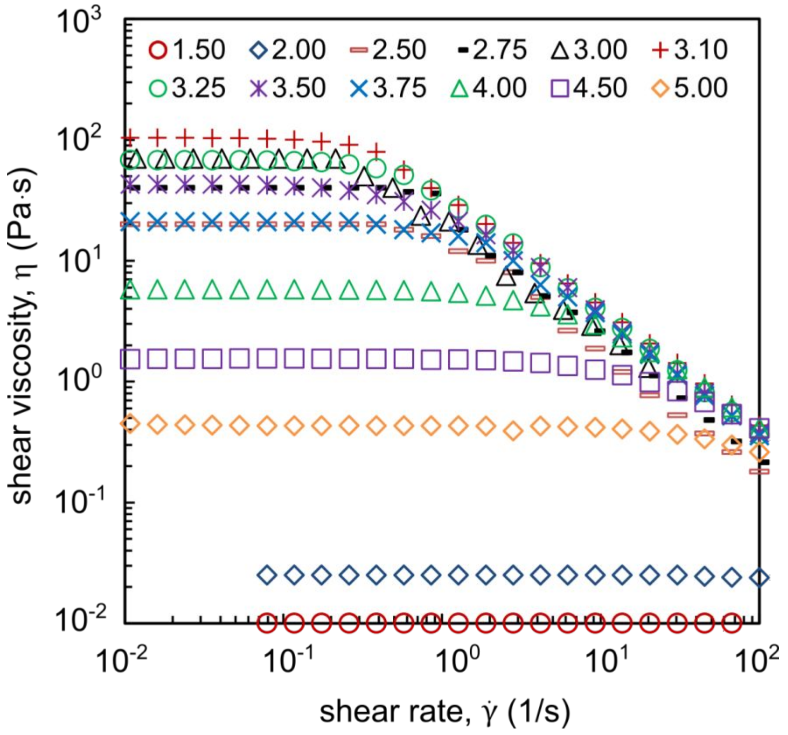

Fig. S2. Viscosity curves of CAPB/SDBS solutions with the addition of 0.1M concentration of Ca^{2+} ions

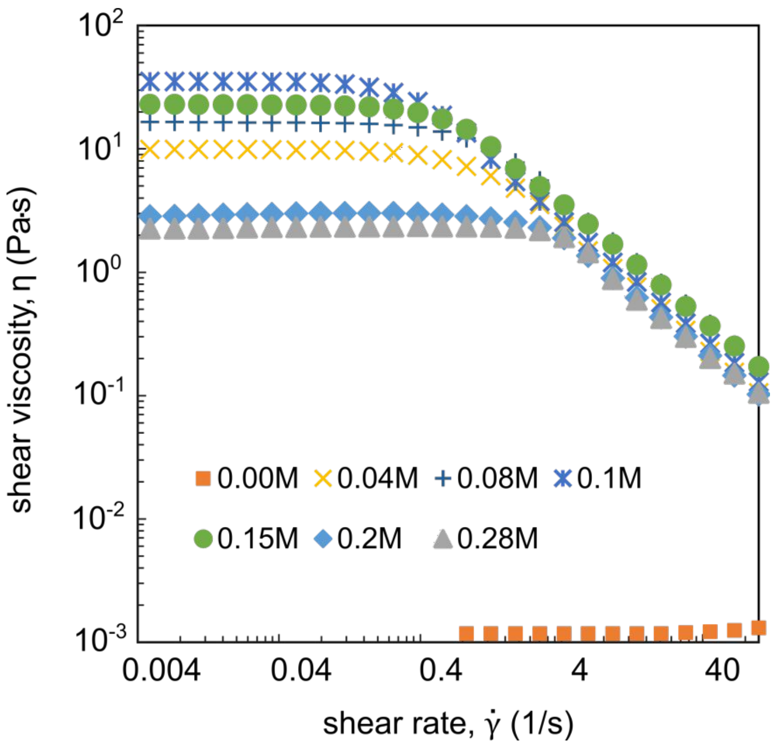

Fig. S3. Viscosity curves of CAPB/SDBS solutions with a constant molar ratio of 3.5 and varying Mg^{2+} molar concentration

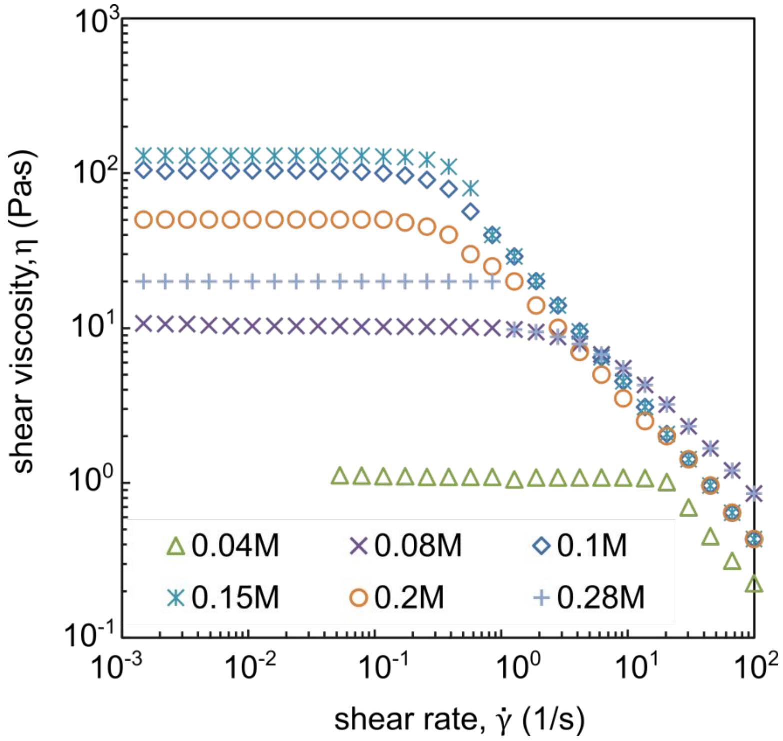

Fig. S4. Viscosity curves of CAPB/SDBS solutions with a constant molar ratio of 3.5 and varying Ca^{2+} ion molar concentration
